# Divergent early host transcriptional responses to H5N8 highly pathogenic avian influenza virus in chickens and ducks

**DOI:** 10.1016/j.psj.2026.107242

**Published:** 2026-06-11

**Authors:** Joanna Sajewicz-Krukowska, Katarzyna Domańska-Blicharz, Karolina Tarasiuk, Krzysztof Śmietanka, Barbara Marzec-Kotarska

**Affiliations:** a Department of Virology and Viral Animal Diseases, National Veterinary Research Institute, 57 Partyzantów Avenue, 24-100 Puławy, Poland; b Department of Clinical Pathomorphology, The Medical University of Lublin, Poland

**Keywords:** H5N8, Highly pathogenic avian influenza virus, Chickens, Ducks, Transcriptomics

## Abstract

Highly pathogenic avian influenza viruses (HPAIVs) of the H5N8 subtype have caused severe outbreaks in poultry and wild birds worldwide. Disease outcome differs markedly between avian species: chickens typically develop acute infection with high mortality, whereas ducks often exhibit milder or delayed disease. The molecular basis underlying these species-specific differences in early host responses remains incompletely understood.

To characterize early transcriptional responses to HPAIV infection, RNA sequencing (RNA-seq) was performed on lung tissues from chickens and ducks experimentally infected with H5N8 HPAIV at 24 hours post-inoculation (hpi). Differential gene expression analysis and functional enrichment analyses were conducted, and selected host genes were validated by quantitative real-time PCR.

At 24 hpi, a comparable number of differentially expressed genes (DEGs) was identified in chickens and ducks; however, the organization of the transcriptional response differed markedly between species. Chickens exhibited a pathway-centered transcriptional profile dominated by innate immune and inflammatory signaling, whereas ducks displayed a more distributed and balanced transcriptional response spanning multiple functional categories. Functional enrichment analyses revealed strong activation of interferon- and cytokine-associated pathways in chickens, while transcriptional changes in ducks were dispersed across processes related to RNA metabolism, antiviral regulation, and cellular homeostasis. Among species-specific differentially expressed genes, MOV10L1 showed opposite regulation in these bird species.

These findings demonstrate that early host responses to H5N8 HPAIV infection differ between chickens and ducks not only in gene identity but also in transcriptional organization. The distributed response architecture observed in ducks may contribute to controlled antiviral responses with limited immunopathology. This study provides a comparative transcriptomic framework for understanding species-specific host responses to HPAIV infection in poultry.

## Introduction

Avian influenza viruses (AIVs), members of the *Orthomyxoviridae* family, represent a continuous threat to animal and human health. Based on their pathogenicity in chickens, AIVs are classified as low pathogenic avian influenza (LPAI) or highly pathogenic avian influenza (HPAI) viruses. Subtyping is determined by the combination of surface glycoproteins hemagglutinin (HA) and neuraminidase (NA), with H5 and H7 subtypes most frequently associated with severe disease. Due to the high error rate of the viral RNA polymerase, H5 and H7 LPAIVs may evolve into highly pathogenic forms, resulting in devastating outbreaks in poultry and occasional spillover into mammals, including humans (Neumann et al., 2007; Webster et al., 2014).

H5N8 clade 2.3.4.4b HPAIV has emerged as a major global concern over the past decade. Large-scale outbreaks during 2016–2017 and again in 2020–2021 led to the culling of millions of poultry and unprecedented mortality among wild birds across Europe, Asia, and North America (Lee at al., 2016; Global Consortium for H5N8 and Related Influenza Viruses, 2019; Rafique et al., 2013). Waterfowl play a central role in the ecology of avian influenza viruses by facilitating long-distance dissemination and genetic reassortment; however, disease severity in these species can vary depending on viral strain, host species, and age at infection (Verhagen et al., 2021; Beerens et al., 2021). Since 2021, H5N1 has largely replaced H5N8 as the dominant HPAIV subtype, causing the largest recorded HPAI epidemic in Europe and spreading to the Americas, with increasing reports of spillover into mammalian species (Dziadek et al., 2024; Prosser et al., 2024; Abdelwhab et al., 2023).

Disease outcome following HPAIV infection differs markedly between avian species. Chickens are highly susceptible and frequently develop rapid, systemic disease with high mortality, whereas ducks often exhibit milder or delayed clinical manifestations, although severe disease has also been reported for certain H5N8 strains. These interspecies differences have been attributed to multiple factors, including host–virus coevolution, innate immune sensing, sialic acid receptor distribution, and physiological traits such as body temperature regulation (Wille et al., 2022; Puranik et al., 2020). A well-characterized example is the absence of retinoic acid–inducible gene I (RIG-I) in chickens, in contrast to ducks, which possess a functional RIG-I pathway and mount a controlled type I interferon response that can limit viral replication without excessive inflammation (Karpala et al., 2011; El-Shall et al., 2023; Bessière et al., 2022).

Recent transcriptomic studies have demonstrated that chickens and ducks differ not only in the magnitude but also in the organization of their early host responses to AIV infection (Smith et al., 2015; Naguib et al., 2023). Chickens typically exhibit strong activation of interferon-stimulated genes and inflammatory pathways, whereas ducks display a more distributed transcriptional response involving antiviral, metabolic, and regulatory processes. However, the molecular features underlying these distinct response patterns during the early phase of H5N8 HPAIV infection remain incompletely characterized.

In this study, we used RNA sequencing (RNA-seq) to compare early lung transcriptomic responses in chickens and ducks experimentally infected with H5N8 HPAIV at 24 hours post-inoculation. Our aim was to identify species-specific patterns of gene expression and pathway organization that characterize early host responses to infection. By focusing on comparative transcriptional profiles rather than clinical outcomes, this work seeks to provide insight into molecular features associated with species-specific organization of early host responses to HPAIV in poultry species.

## Materials and methods

### Virus

The H5N8 highly pathogenic avian influenza virus (HPAIV) strain A/herring gull/Poland/MB082B/2016 (clade 2.3.4.4b) was isolated during the 2016–2017 epizootic in Poland (Świętoń et al., 2018). The virus was propagated in the allantoic cavities of 9–11-day-old specific pathogen–free (SPF) embryonated chicken eggs (Valo Biomedia, Osterholz-Scharmbeck, Germany) at 37°C for 48 h. Allantoic fluids were harvested after chilling, clarified by low-speed centrifugation, aliquoted, and stored at −80°C until use. Viral titers were determined as the 50 % egg infectious dose (EID₅₀/mL) according to the Reed and Muench method (Reed et al., 1938). The genome sequence of this strain is available in the GISAID database (Accession ID: EPI_ISL_15430045).

### Experimental animals and infection

Three-week-old Ross 308 chickens and Pekin ducks were obtained from local hatcheries and housed at the National Veterinary Research Institute (NVRI, Poland). Before the experiment, birds were confirmed negative for avian influenza virus infection by molecular and serological testing. For each species, birds were randomly assigned to infected (n = 5) or control (n = 5) groups.

At three weeks of age, birds in the infected groups were inoculated via the intraocular and intranasal routes with 10⁶ EID₅₀ per 0.1 mL of virus suspension per bird. Control birds received sterile phosphate-buffered saline (PBS) using the same routes. Animals were housed in separate biocontainment rooms under BSL-3+ conditions with ad libitum access to food and water.

Birds were monitored for 24 h post-inoculation (hpi). At 24 hpi, tracheal and cloacal swabs were collected, after which birds were humanely euthanized and lung tissues were harvested for downstream analyses.

### RNA extraction and sequencing

Lung tissues were homogenized in RLT buffer supplemented with β-mercaptoethanol using an MP FastPrep-24 Tissue and Cell Homogenizer (MP Biomedicals, USA). Total RNA was extracted from lung homogenates and from tracheal and cloacal swabs using the RNeasy Mini Kit (Qiagen, Germany) according to the manufacturer’s instructions. RNA integrity was assessed using a Bioanalyzer, and only samples with an RNA integrity number (RIN) > 7 were used for sequencing.

A total of 20 cDNA libraries were prepared (Macrogen Europe, Amsterdam, the Netherlands) using the TruSeq Stranded mRNA LT Kit (Illumina, San Diego, CA, USA) and sequenced on the Illumina NovaSeq 6000 platform to generate 150-bp paired-end reads.

### Quantification of viral RNA

Viral RNA levels in tracheal and cloacal swabs, as well as in lung tissues were assessed by quantitative real-time PCR (qRT-PCR) targeting the influenza A virus matrix (M) gene. RNA extracted as described above was subjected to one-step qRT-PCR using primers and probes specific for the M gene, as previously described by Spackman et al. (2002). Reactions were performed on a 7500 Real-Time PCR System (Applied Biosystems, Foster City, CA, USA).

Viral RNA detection was used to confirm successful infection in inoculated birds at 24 h post-inoculation and was not intended for comparative analysis of viral replication between species.

### Bioinformatic and statistical analysis

Raw sequencing reads were assessed for quality using FastQC (Andrews, 2010) and MultiQC (Ewels et al., 2016), trimmed with Cutadapt (Martin, 2011), and aligned to the *Gallus gallus* (GRCg6a) and *Anas platyrhynchos* (CAU_duck1.0) reference genomes using STAR (Dobin et al., 2013). Differential gene expression analysis was performed using DESeq2 (Love et al., 2014), edgeR (Robinson et al., 2010), and voom (Law et al., 2014). Genes were considered differentially expressed if they met the criteria of |log₂ fold change| ≥ 2 and a false discovery rate (FDR)–adjusted p-value < 0.05.

Functional enrichment analysis was conducted using g:Profiler (Raudvere et al., 2019), including Gene Ontology (GO) categories (Biological Process, Molecular Function, and Cellular Component) as well as Kyoto Encyclopedia of Genes and Genomes (KEGG) pathways. Enrichment results with p < 0.05 were considered significant. Differential expression results were considered robust when supported by concordant findings across methods.

### Data availability

The raw RNA-seq data generated in this study have been submitted to the Sequence Read Archive (SRA) under accession number SUB15644184 and will be publicly released on 24 February 2027.

### Ethical considerations

All animal experiments were approved by the Local Ethics Committee (reference number 1/2020) and conducted in accordance with EU Directive 2010/63/EU on the protection of animals used for scientific purposes.

### qRT-PCR validation of selected host genes

To validate RNA-seq results, one-step quantitative real-time PCR (qRT-PCR) was performed for a subset of host genes showing significant differential expression in the transcriptomic analysis, as listed in Table 1. Gene selection was based on fold change, statistical significance, and representation of major functional categories identified by RNA-seq. In ducks, *DDX58* and *MOV10L1* were analyzed, while in chickens *IFIT5* and *MX1* were selected.

**Table 1:** Primers and probes used for qRT-PCR analysis. Primers and probes were designed based on gene sequences of chickens (*Gallus gallus*, abbreviated ch) and ducks (*Anas platyrhynchos domesticus*, abbreviated du). Sequences are shown in the 5′–3′ orientation. Commercial TaqMan assays were obtained from Thermo Fisher Scientific, while other primers and probes were either retrieved from published studies or designed in this study.

**Target gene**	**Sequence (5′−3′)**	**Reference**
*duGAPDH*	F: AAATTGTCAGCAATGCCTCTTG	Saito et al*.,* 2018
	R: TGGCATGGACAGTGGTCATAA	
	Probe: ACCACCAACTGCCTGGCGCC	
*duDDX58*	F: GGAGAGCAGGATATGTAGAG	
	R: GGTCAGGTAGGATAAAGCATC	
	Probe: TCCGCAGGTGTTCAGTGCAAATGAAA	
*duMOV10L1*	F: CCTACTGCTGCTTACACCTATT	Author-designed (this study)
	R: CACTCTGGCCTCTACCATTTC	
	Probe: AAGTGTGCTCATGCTCTCCTTGAGG	
*chGAPDH*	N/A	Thermo Fisher, Gg03346982_m1
*chIFIT5*	F: AAAAGAAGGCAAATCATGAGTACC	Cong et al., 2013
	R: TGATCCTCTATTGATTCTTCCAGAC	
	Probe: AATTCCTTGAAGAACTCCCTGCTGC	
*chMX1*	N/A	Thermo Fisher, Gg03337834_m1

GAPDH served as the reference gene for normalization in both species. Primers and probes were obtained from published sources, commercial assays, or designed by the authors in this study (Saito et al., 2018; Cong et al., 2013). qRT-PCR reactions were carried out using the QuantiTect Probe RT-PCR Kit (Qiagen, Germany) on a 7500 Real-Time PCR System (Applied Biosystems, Foster City, CA, USA). Relative expression levels were calculated using the 2^−ΔΔCt method (Livak et al., 2001).

## Results

### Clinical outcome

Following experimental inoculation with H5N8 HPAIV, all birds were monitored for 24 h before scheduled euthanasia and sample collection. During this observation period, no severe clinical signs or extensive gross pathological lesions were recorded in either species. In chickens, mild behavioral changes, including reduced activity and clustering, were observed, and splenic and pulmonary congestion was detected in one individual at necropsy. Ducks remained clinically unremarkable throughout the 24 h observation window.

Viral RNA was detected in tracheal and cloacal swabs collected at 24 h post-inoculation (hpi), confirming successful infection in both species.

The limited clinical manifestation observed in both species is consistent with the early time point selected for analysis and the experimental design, which aimed to capture early host responses prior to the development of advanced disease.

### Sequencing output and differential gene expression analysis

RNA sequencing generated a total of 184,235,140 paired-end reads from chicken lung samples (n = 10; 5 infected and 5 control) and 173,211,232 paired-end reads from duck lung samples (n = 10; 5 infected and 5 control). On average, more than 92 % of reads from chicken samples and more than 85 % from duck samples mapped uniquely to their respective reference genomes. Detailed sequencing and alignment metrics are provided in Table 2.

**Table 2:** Sequencing and alignment statistics. Summary of sequencing and alignment results for chicken and duck samples, including total reads, average read length, uniquely mapped reads, and mapping percentage. Sample IDs containing KA refer to ducks (Polish: kaczka), while those containing KU refer to chickens (kura).

**Sample name**	**Number of input reads (pairs)**	**Average input read length**	**Uniquely mapped reads count**	**Uniquely mapped reads %**
**397KA_H5N8**	15 567 901	290	13 320 790	85,57 %
**398KA_H5N8**	18 492 440	292	16 096 166	87,04 %
**400KA_H5N8**	14 901 619	292	13 060 984	87,65 %
**401KA_H5N8**	15 642 235	292	13 735 227	87,81 %
**402KA_H5N8**	19 315 590	293	17 041 709	88,23 %
**399KA_PBS**	21 369 300	293	18 823 807	88,09 %
**405KA_PBS**	16 944 348	294	15 109 433	89,17 %
**407KA_PBS**	19 333 402	292	16 994 217	87,90 %
**409KA_PBS**	16 272 138	292	14 008 503	86,09 %
**411KA_PBS**	15 372 259	293	13 215 860	85,97 %
**536_KU_PBS**	14 833 869	292	14 015 315	94,48 %
**537_KU_PBS**	19 405 778	290	18 325 018	94,43 %
**538_KU_PBS**	18 509 457	290	17 443 345	94,24 %
**539_KU_PBS**	21 559 015	292	20 395 730	94,60 %
**540_KU_PBS**	17 451 304	284	16 405 825	94,01 %
**541_KU_H5N8**	20 703 011	289	19 658 537	94,95 %
**542_KU_H5N8**	18 031 702	290	17 114 784	94,91 %
**543_KU_H5N8**	15 170 917	290	14 005 602	92,32 %
**545_KU_H5N8**	18 242 075	289	17 201 369	94,30 %
**546_KU_H5N8**	20 328 012	289	19 184 399	94,37 %

At 24 hpi, comparison of infected and control groups identified 440 differentially expressed genes (DEGs) in chickens, of which 431 were upregulated and 9 downregulated. In ducks, 464 DEGs were detected, including 346 upregulated and 118 downregulated genes. Although the total number of DEGs was comparable between species, the distribution of expression changes differed markedly. Chickens showed a predominance of upregulated genes, whereas ducks displayed a more balanced pattern comprising both up- and downregulated transcripts.

**Fig. 1:**
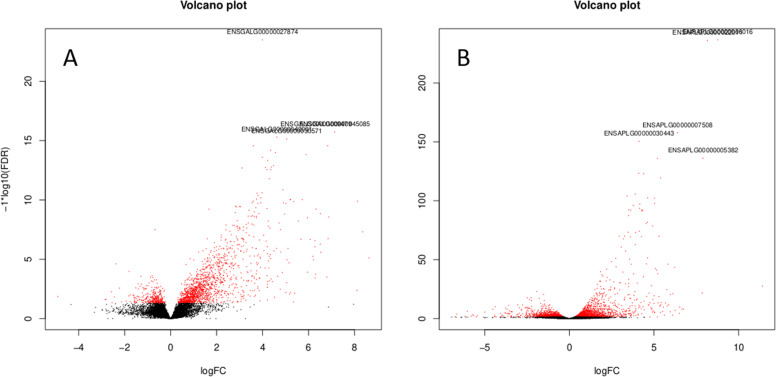
Volcano plots of differentially expressed genes (DEGs) in chickens (A) and ducks (B). Each point represents a gene, plotted by log₂ fold change (x-axis) and –log₁₀(FDR) (y-axis). Red points indicate statistically significant DEGs (FDR < 0.05).

**Fig. 2:**
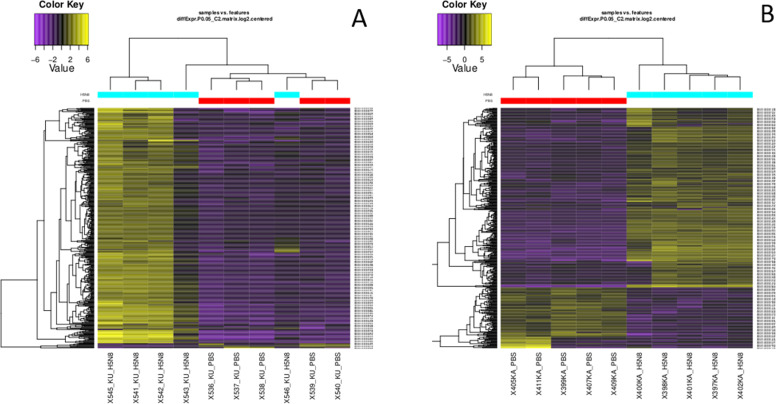
Heatmap of differentially expressed genes (DEGs) in chicken (A) and duck (B) lungs at 24 hpi. Rows represent genes, and columns represent individual samples. H5N8-infected samples form distinct clusters compared to PBS controls, confirming infection-induced transcriptional reprogramming.

These differences were visualized using volcano plots and hierarchical clustering heatmaps (Fig. 1, Fig. 2), which demonstrated a strong infection-associated transcriptional shift in infected samples relative to controls in both species, with distinct expression profiles between chickens and ducks. Complete DEG lists are provided in Supplementary files 1 and 2.

### Functional enrichment analysis

Gene Ontology (GO) analysis demonstrated that in chickens, the most significantly enriched biological process (BP) terms were predominantly associated with innate antiviral and inflammatory responses, including response to virus, type I interferon signaling pathway, cytokine-mediated signaling pathway, and defense response to other organisms. Additional enrichment was observed for processes related to programmed cell death and cellular stress responses.

In ducks, enriched GO biological process terms were more heterogeneous and encompassed RNA metabolic processes, regulation of viral replication, innate immune response, regulation of apoptotic processes, as well as pathways associated with cellular homeostasis and metabolic adaptation. Compared with chickens, enriched terms in ducks were distributed across a broader range of functional categories.

**Fig. 3:**
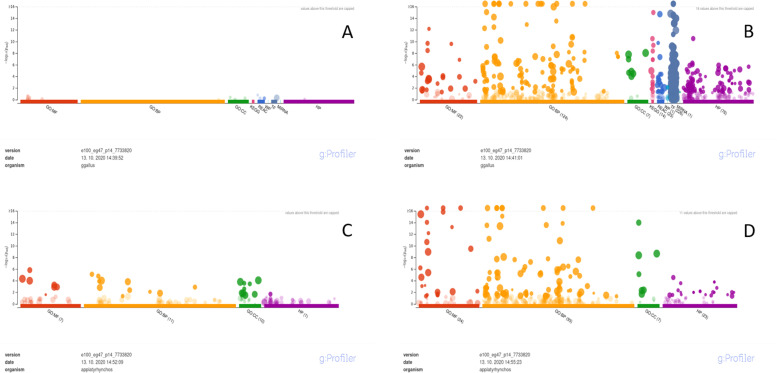
Gene Ontology (GO) and pathway enrichment analysis of upregulated genes in chickens (A-B) and ducks (C-D). Panels show biological processes enriched in chickens H5N8-infected (A) and PBS-inoculated controls (B) as well as in ducks H5N8-infected (C) and PBS-inoculated controls (D). Enriched terms are color-coded according to annotation sorce as follows:: Biological Process (BP) in yellow, Molecular Function (MF) in red, and Cellular Component (CC) in green, KEGG pathways in pink, Reactome pathways (REAC) in blue, transcription factor target gene sets (TF) in blue-grey, and Hallmark/HP-related gene sets in purple. Each dot represents an enriched term plotted accordin to -log_10_ adjusted p-value. Only statistically significant terms are shown.

Analysis of molecular function (MF) categories revealed enrichment of cytokine and chemokine activity, receptor binding, and nucleic acid binding in chickens, whereas ducks additionally showed enrichment of hydrolase and transferase activities. Within the cellular component (CC) domain, enriched categories in both species included extracellular and membrane-associated components, while ducks also exhibited enrichment of mitochondrial and cytoplasmic components (Fig. 3).

### KEGG pathway enrichment

Kyoto Encyclopedia of Genes and Genomes (KEGG) pathway analysis identified significant enrichment in chickens for pathways including influenza A, cytokine–cytokine receptor interaction, Toll-like receptor signaling, NOD-like receptor signaling, necroptosis, and herpes simplex virus 1 infection. In contrast, no KEGG pathways reached the significance threshold in ducks. This observation indicates that, at 24 h post-inoculation (hpi), differentially expressed genes in ducks were not clustered within canonical immune signaling pathways but were instead distributed across multiple functional categories.

A complete list of GO and KEGG enrichment results is provided in Supplementary files 3–6.

### Notable species-specific differentially expressed genes

In chickens, highly upregulated genes included classical interferon-stimulated genes (ISGs) such as *MX1, RSAD2, OASL, CCL19*, and *IFIT5*, which are commonly associated with antiviral interferon responses. In ducks, upregulated genes included *DDX58, EPSTI1, MOV10L1, ACOD1, BCL2L15*, and *PLAC8*, which together represent a range of antiviral, RNA regulatory, and cellular stress–related functions.

Among these genes, *MOV10L1* displayed opposite regulation between species, being upregulated in ducks and downregulated in chickens. This pattern represents one example of species-specific gene regulation observed in the transcriptomic dataset.

### qRT-PCR validation

**Fig. 4:**
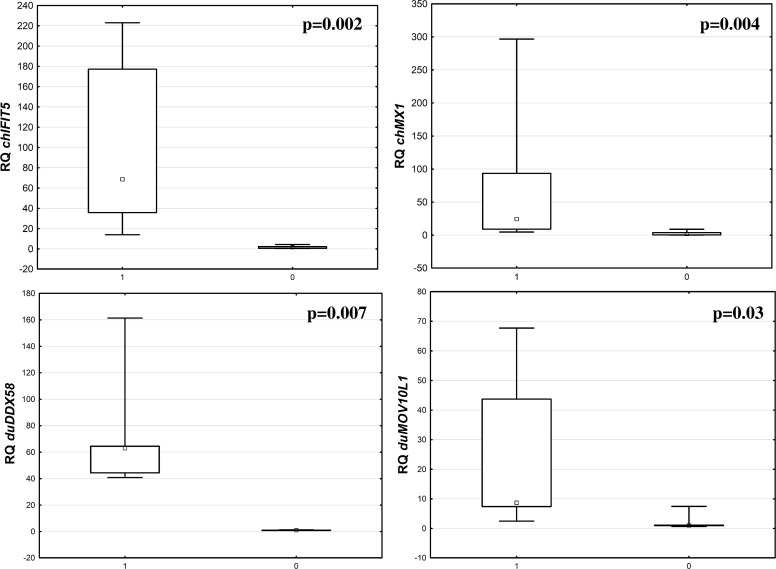
Validation of selected DEGs by quantitative real-time PCR (qRT-PCR). Relative expression levels (RQ) of selected differentially expressed genes (DEGs) in lung tissues of chickens and ducks infected with H5N8 highly pathogenic avian influenza virus (HPAIV) at 24 hours post-inoculation. Gene expression was normalized to *GAPDH* and expressed as fold change relative to PBS-inoculated controls. Data are presented as medians with interquartile ranges (IQR) based on biological replicates (n = 5)**.** On the x-axis, “1″ denotes H5N8-infected groups and “0″ denotes PBS-inoculated controls. Prefixes ch and du preceding gene symbols indicate genes originating from chicken and duck sequences, respectively. The qRT-PCR results confirmed the direction and magnitude of differential expression observed in the RNA-seq analysis.

Quantitative real-time PCR (qRT-PCR) was performed to validate RNA-seq results for a subset of selected genes. In chickens, expression of *IFIT5* and *MX1* was assessed, while in ducks *DDX58* and *MOV10L1* were analyzed, using *GAPDH* as the reference gene (Fig. 4). qRT-PCR results confirmed the direction of differential expression observed in the RNA-seq analysis for all tested genes. Following H5N8 infection, *IFIT5* and *MX1* were strongly upregulated in chickens, whereas *DDX58* and *MOV10L1* were upregulated in ducks.

## Discussion

### Species-specific organization of early host responses to H5N8 HPAIV

This study provides molecular evidence for divergent early host responses of chickens and ducks to H5N8 HPAIV infection. Although a comparable number of differentially expressed genes was identified at 24 h post-inoculation, the overall organization of these responses differed markedly between species. Chickens exhibited a pathway-centered transcriptional profile dominated by innate immune and inflammatory signaling, whereas ducks displayed a more distributed and balanced transcriptional response spanning multiple functional categories.

The contrasting enrichment patterns observed in GO and KEGG analyses further support the notion that chickens and ducks differ not in the magnitude of their transcriptional responses but in their organizational structure. In chickens, enrichment of multiple interconnected immune pathways reflects coordinated activation of canonical antiviral signaling cascades. In ducks, the lack of KEGG pathway enrichment despite clear transcriptional changes suggests a response distributed across diverse cellular processes rather than concentrated within defined immune pathways. Our data indicate that chickens mount a rapid and strongly inflammatory transcriptional response to HPAIV infection as early as 24 hours post-inoculation, whereas ducks exhibit a more controlled and distributed antiviral transcriptional program, which may contribute to species-specific differences in disease outcome.

The 24 h post-inoculation time point was selected to capture early transcriptional divergence while minimizing confounding effects of advanced clinical disease. Accordingly, overt clinical signs were limited in both species at this stage. Nevertheless, the transcriptional patterns observed here likely reflect early host response programs that precede the development of more pronounced pathological manifestations described at later stages of HPAIV infection in chickens, as reported in previous studies.

While differences between chickens and ducks in their responses to avian influenza viruses have been documented previously, the present study extends these observations by providing a comparative transcriptomic snapshot of early host response organization during H5N8 HPAIV infection. Among the genes displaying species-specific regulation, *MOV10L1* emerged as one example of differential expression between chickens and ducks, warranting further investigation in the context of early host responses.

### Inflammatory and interferon-driven responses in chickens

In chickens, enrichment of Toll-like receptor (TLR) and NOD-like receptor signaling pathways, cytokine–cytokine receptor interactions, and necroptosis reflects strong activation of innate immune signaling during early H5N8 HPAIV infection. Recognition of viral RNA through these pathways activates downstream cascades involving NF-κB and interferon regulatory factors, resulting in robust induction of type I interferon responses (Wurzer et al., 2004; Orzalli et al., 2017).

Consistent with this, classical interferon-stimulated genes (ISGs) such as *MX1, RSAD2*, and *OASL* were among the most strongly upregulated transcripts in infected chickens. Similar transcriptional profiles have been reported previously in chickens infected with highly pathogenic avian influenza viruses and are characteristic of interferon-driven responses (Karpala et al., 2011; Kuchipudi et al., 2014). While activation of these pathways is essential for antiviral defense, previous studies have shown that excessive or poorly regulated inflammatory signaling can be associated with immunopathology during HPAIV infection in both avian and mammalian hosts (Cheung et al., 2002; Kobasa et al., 2007; Teijaro et al., 2011).

In addition, enrichment of necroptosis-related pathways suggests activation of programmed cell death mechanisms that have been implicated in the amplification of inflammatory responses during influenza virus infection. Although necroptosis may contribute to viral control, previous studies indicate that it can also exacerbate inflammation depending on the cellular context (Wurzer et al., 2004; Orzalli et al., 2017). Together, these observations are consistent with a transcriptional response dominated by innate immune and inflammatory signaling in chickens at an early stage of H5N8 infection.

### Distributed transcriptional responses in ducks

In contrast to the pathway-centered response observed in chickens, ducks exhibited a transcriptional profile characterized by a broader distribution of differentially expressed genes across multiple functional categories. At 24 h post-inoculation, no significant enrichment of canonical immune signaling pathways was detected in ducks, despite clear evidence of infection. Instead, enriched Gene Ontology terms were associated with RNA metabolic processes, regulation of viral replication, cellular stress responses, and apoptosis.

This distributed transcriptional organization suggests that early host responses in ducks may rely less on strong activation of individual signaling cascades and more on coordinated modulation of diverse cellular processes. Importantly, the absence of pathway-level enrichment does not imply a weak response but rather indicates a different mode of transcriptional organization during early infection. Similar patterns have been reported previously in transcriptomic studies of ducks infected with avian influenza viruses and have been proposed to reflect species-specific differences in immune regulation (Naguib et al., 2023; Kuchipudi et al., 2014).

At the gene level, several differentially expressed genes in ducks are consistent with early viral RNA sensing and antiviral regulation. Among these, *DDX58 (RIG-I)* was upregulated, in line with the presence of a functional RIG-I pathway in ducks that is absent in chickens and its established role in cytosolic recognition of influenza viral RNA (Karpala et al., 2011; Rehwinkel et al., 2020). Additional duck DEGs, including *EPSTI1, ACOD1*, and *PLAC8*, have previously been associated with modulation of innate immune signaling, cellular stress responses, or metabolic adaptation during viral infection (Naguib et al., 2023; Michelucci et al., 2013). Their concurrent regulation may reflect coordinated fine-tuning of cellular responses rather than strong activation of individual immune cascades (Naguib et al., 2023).

Notably, ducks displayed a substantial proportion of downregulated genes relative to chickens. This balanced pattern of up- and down-regulation may reflect coordinated transcriptional reprogramming, involving not only activation of antiviral responses but also modulation of baseline cellular functions. Similar distributed transcriptional architectures have been reported in other duck influenza studies and may represent a characteristic feature of early host responses in this species (Naguib et al., 2023). Such distributed transcriptional organization may represent an early regulatory strategy that limits excessive inflammatory signaling while maintaining antiviral control.

### MOV10L1 as a species-specific transcriptional feature

Among genes showing species-specific regulation, *MOV10L1* displayed opposite expression patterns in ducks and chickens. *MOV10L1* encodes an RNA helicase best known for its role in PIWI (P-element–induced wimpy testis)–interacting RNA (piRNA) biogenesis and transposon silencing and has not been directly implicated in influenza virus infection (Naguib et al., 2023; Świętoń et al., 2018). Its differential regulation in the present study therefore represents an intriguing transcriptional feature associated with early host responses rather than evidence of a demonstrated antiviral function.

Previous studies of the related paralog *MOV10 (Moloney leukemia virus 10)* have shown that RNA helicases can participate in antiviral responses by modulating RNA stability and innate immune signaling (Cuevas et al., 2016; Nawaz et al., 2022). Whether *MOV10L1* fulfills similar or distinct functions in somatic tissues during viral infection remains unknown. Given the lack of functional data in the current study, the biological significance of *MOV10L1* regulation during HPAIV infection should be interpreted with caution and warrants further investigation.

### Evolutionary and ecological considerations

The contrasting transcriptional response architectures observed in chickens and ducks should be interpreted in the context of host–pathogen coevolution. Ducks and other waterfowl have coexisted with avian influenza viruses over extended evolutionary timescales, shaping immune systems that differ fundamentally from those of galliform birds (Evseev et al., 2019; Barber et al., 2010). Although Pekin ducks were used in this study, their responses likely reflect ancestral traits inherited from wild waterfowl such as mallards (*Anas platyrhynchos*), which are recognized natural reservoirs of avian influenza viruses (Prosser et al., 2024).

Such evolutionary history may have favored immune response strategies that rely on distributed transcriptional regulation rather than strong pathway-level activation. The species-specific transcriptional features identified here, including differences in pathway organization and regulation of RNA-associated genes, may represent components of these long-term adaptations (Evseev et al., 2019). However, extrapolation from early transcriptional responses to ecological or epidemiological outcomes should be made cautiously.

### Limitations and future directions

This study has several limitations. Analysis was restricted to a single early time point, which does not capture the temporal dynamics of host responses during infection. Transcriptomic profiling was limited to lung tissue, although HPAIV infection is systemic. In addition, sample size and qRT-PCR validation were constrained by the requirements of high-containment animal experiments.

Future studies incorporating longitudinal sampling, additional tissues, and functional assays will be required to determine how early transcriptional organization relates to subsequent disease progression. In particular, experimental manipulation of candidate genes identified here, including *MOV10L1*, may help clarify their biological relevance during influenza virus infection. Integration of transcriptomic data with proteomic, metabolomic, or single-cell approaches could further refine our understanding of host–virus interactions in different avian species.

## Conclusions

Chickens and ducks exhibit fundamentally different early transcriptional responses to H5N8 highly pathogenic avian influenza virus infection. Although both species respond robustly at the level of gene expression, the organization of these responses differs markedly, with chickens displaying a pathway-centered inflammatory profile and ducks showing a more distributed transcriptional architecture. These findings highlight species-specific differences in early host response organization and provide a comparative transcriptomic framework for understanding avian influenza virus infections in poultry species.

## Funding

This research was funded by the National Science Centre in Poland (Grant No. 2019/03/X/NZ2/00577).

## Institutional review board statement

Animal experiments were performed in agreement with the rules in place in the EU (Directive 2010/63/UE) and approved by the Local Ethics Commission.

## Informed consent statement

Not applicable.

## Data availability statement

The raw data generated in RNA-seq study were submitted to the SRA database (https://www.ncbi.nlm.nih.gov/sra) (accessed on 2025-10-02) under accession number SUB15644184 (release date: 2027-02-24).

## Author contributions

Conceptualization, J.S.-K.; Methodology, J.S.-K.; Validation, J.S.-K. and B.M.-K.; Formal analysis, J.S.-K.; Investigation, J.S.-K. and K.T.; Data curation, J.S.-K.; Writing – original draft, J.S.-K.; Writing – review & editing, J.S.-K., B.M.-K., K.Ś., and K.D.-B.; Visualization, J.S.-K.; Supervision, J.S.-K.; Funding acquisition, J.S.-K.

## Disclosures

The authors declare that they have no known competing financial interests or personal relationships that could have appeared to influence the work reported in this paper.

## Appendix Supplementary materials

Supplementary files: Supplementary file 1: Complete list of differentially expressed genes (DEGs) identified in lung tissues of chickens (*Gallus gallus*) infected with H5N8 highly pathogenic avian influenza virus (HPAIV) at 24 hours post-inoculation, Supplementary file 2: Complete list of differentially expressed genes (DEGs) identified in lung tissues of ducks (*Anas platyrhynchos domesticus*) infected with H5N8 highly pathogenic avian influenza virus (HPAIV) at 24 hours post-inoculation, Supplementary file 3: Gene Ontology (GO) and Kyoto Encyclopedia of Genes and Genomes (KEGG) enriched terms associated with upregulated genes (H5N8-UP) in lungs of chickens (*Gallus gallus*) infected with H5N8 highly pathogenic avian influenza virus (HPAIV) at 24 hours post-inoculation, Supplementary file 4: Gene Ontology (GO) and Kyoto Encyclopedia of Genes and Genomes (KEGG) enriched terms associated with upregulated genes (PBS-UP) in lungs of chickens (*Gallus gallus*) from the PBS control group compared to H5N8-infected birds at 24 hours post-inoculation, Supplementary file 5: Gene Ontology (GO) and Kyoto Encyclopedia of Genes and Genomes (KEGG) enriched terms associated with upregulated genes (H5N8-UP) in lungs of ducks (*Anas platyrhynchos domesticus*) infected with H5N8 highly pathogenic avian influenza virus (HPAIV) at 24 hours post-inoculation, Supplementary file 6: Gene Ontology (GO) and Kyoto Encyclopedia of Genes and Genomes (KEGG) enriched terms associated with upregulated genes (PBS-UP) in lungs of ducks (*Anas platyrhynchos domesticus*) from the PBS control group compared to H5N8-infected birds at 24 hours post-inoculation. Image, application 1

## Footnotes

Supplementary material associated with this article can be found, in the online version, at doi:10.1016/j.psj.2026.107242.
